# *Wheat streak mosaic virus* P1 Binds to dsRNAs without Size and Sequence Specificity and a GW Motif Is Crucial for Suppression of RNA Silencing

**DOI:** 10.3390/v11050472

**Published:** 2019-05-24

**Authors:** Adarsh K. Gupta, Satyanarayana Tatineni

**Affiliations:** 1 Department of Plant Pathology, University of Nebraska-Lincoln, Lincoln, NE 68583, USA; adarsh.bio@gmail.com; 2 United States Department of Agriculture-Agricultural Research Service, Lincoln, NE 68583, USA

**Keywords:** Wheat streak mosaic virus, P1, RNA silencing suppressor, dsRNA binding

## Abstract

*Wheat streak mosaic virus* (WSMV; genus *Tritimovirus*; family *Potyviridae*) is an economically important virus infecting wheat in the Great Plains region of the USA. Previously, we reported that the P1 protein of WSMV acts as a viral suppressor of RNA silencing. In this study, we delineated the minimal region of WSMV P1 and examined its mechanisms in suppression of RNA silencing. We found that the 25 N-terminal amino acids are dispensable, while deletion of a single amino acid at the C-terminal region completely abolished the RNA silencing suppression activity of P1. Electrophoretic mobility shift assays with in vitro expressed P1 revealed that the P1 protein formed complexes with green fluorescent protein-derived 180-nt dsRNA and 21 and 24-nt ds-siRNAs, and WSMV coat protein-specific 600-nt dsRNA. These data suggest that the P1 protein of WSMV binds to dsRNAs in a size- and sequence-independent manner. Additionally, in vitro dicing assay with human Dicer revealed that the P1 protein efficiently protects dsRNAs from processing by Dicer into siRNAs, by forming complexes with dsRNA. Sequence comparison of P1-like proteins from select potyvirid species revealed that WSMV P1 harbors a glycine-tryptophan (GW) motif at the C-terminal region. Disruption of GW motif in WSMV P1 through W303A mutation resulted in loss of silencing suppression function and pathogenicity enhancement, and abolished WSMV viability. These data suggest that the mechanisms of suppression of RNA silencing of P1 proteins of potyvirid species appear to be broadly conserved in the family *Potyviridae*.

## 1. Introduction

RNA silencing or post-transcriptional gene silencing (PTGS) is one of the most conserved eukaryotic regulatory pathways of many biological processes such as development, genome stability, and responses to abiotic and antiviral defenses [1,2,3]. RNA silencing is elicited by double-stranded RNA (dsRNA), which mostly originates from replicative intermediates of RNA viruses [4], endogenous molecular triggers such as retrotransposons [5], or pre-micro RNAs [6]. Despite the complexity and diversity of RNA silencing, the basic components are highly conserved among many eukaryotes [7]. These components include RNase III DCL (Dicer-like), which is involved in the dicing of dsRNAs into siRNAs [8], Piwi containing Argonaute (AGO-PIWI), which is the effector protein of siRNA-induced silencing complex (siRISC) that mediates target hydrolysis [9], and RNA-dependent RNA polymerase (RDR), which amplifies RNA silencing into transitivity [10]. Despite strong and specific RNA silencing employed by hosts, viruses not only gain access to but also establish stable and systemic infection in eukaryotes. One of the strategies viruses employ to overcome the host RNA silencing is through encoding viral suppressors of RNA silencing (VSR) [11].

VSRs of different groups of viruses are highly divergent in sequence; however, their RNA silencing pathway targets are functionally conserved. A few silencing suppressors such as coat protein (CP) of *Turnip crinkle virus* or NSs protein of *Tomato spotted wilt virus* bind to long dsRNAs to protect them from the activity of DCL [12,13]. Some suppressors like P1 of *Rice yellow mottle virus* directly block DCL4 to hamper the production of siRNAs [14]. VSRs like P19 of *Cymbidium ring spot virus* or 2b of *Cucumber mosaic virus* indirectly impede the formation of RISC by sequestering PTGS-generated short dsRNAs (ds-siRNAs), which also depletes mobile inducers of systemic silencing [15,16]. However, several VSRs interact directly with AGO at different levels of silencing suppressor’s activity. The P0 protein of *Beet western yellows virus* and the P25 protein of *Potato virus X* (PVX) were mediated ubiquitination of AGO1 for proteolytic degradation [17,18]. Conversely, P1 of *Sweet potato mild mottle virus* (SPMMV) interacts with AGO1 of assembled RISC to block target hydrolysis [19]. In order to suppress amplification of RNA silencing, few VSRs, like viral genome-linked protein (VPg) of *Potato virus A* (PVA), interact with SGS3 protein cofactor of RDR6 [20], which is associated with generation of transacting-siRNAs (ta-siRNAs). However, P6 protein of *Rice yellow stunt virus* directly blocks RDR6 to prevent its ability to generate long dsRNAs for secondary siRNAs production [21]. The ubiquitous nature of VSRs encoded by viruses highlights their paramount importance not only in systemic infection but also to complete the viral life-cycle.

The family *Potyviridae* consists of the largest number of plant RNA viruses that are characterized by a mono- or bi-partite positive-sense single-stranded RNA genome [22]. The genomic RNA encodes a single polyprotein that undergoes posttranslational cleavage into functional viral proteins [23]. Among those viral-encoded proteins, helper component-proteinase (HC-Pro) is involved in RNA silencing suppression function in the genera *Potyvirus* (eg. *Tobacco etch virus*) [24,25] and *Rymovirus* (eg. *Agropyron mosaic virus*) [26]. Interestingly, in addition to HC-Pro, a well characterized VSR, VPg was shown to have VSR function in a species of *Potyvirus*, PVA [20]. In contrast, species of the genus *Ipomovirus* employ P1 as the suppressor of RNA silencing. Ipomoviral species assign their P1 as a VSR, irrespective of the presence or absence of HC-Pro in their genomes. *Cassava brown streak virus* and *Cucurbit vein yellowing virus* (CVYV), which lack HC-Pro, assign their P1 and P1b as VSRs, respectively [27,28]. However, SPMMV uses P1 for VSR activity despite harboring HC-Pro in its genome [19]. Furthermore, P1 proteins of the genera *Tritimovirus* [26] and *Poacevirus* [29] were also shown to function as VSRs. Besides the suppression of RNA silencing function, P1 is one of the virus-encoded proteinases for posttranslational processing of viral polyprotein [23,30]. Furthermore, P1 was also shown to be involved in replication, movement, pathogenicity, and suppression of RNA silencing [25,31,32].

*Wheat streak mosaic virus* (WSMV), the type species of the genus *Tritimovirus* of the family *Potyviridae*, is the most economically important virus infecting wheat in the Great Plains region of the USA [33]. WSMV is transmitted by the eriophyid mite (*Aceria tosichella* Keifer) in a persistent manner [34,35]. The virions of WSMV are long flexuous rods encapsidating a single molecule of positive-sense genomic RNA of 9384 nt [33]. The genomic RNA has a single open reading frame encoding a large polyprotein that undergoes post-translational cleavage into at least 10 mature proteins. One of these proteins, P1, was identified as a suppressor of RNA silencing [26].

In this study, the counter defense mechanisms employed by WSMV P1 to suppress plant-induced RNA silencing were examined. We found that WSMV P1 interacts with dsRNAs in a size and sequence independent manner and protects long dsRNAs from the hydrolytic activity of recombinant Dicer. Additionally, we found that a GW dipeptide, a putative AGO-binding linear peptide motif, in WSMV P1 plays an important role in suppression of RNA silencing.

## 2. Materials and Methods

### 2.1. Preparation of Constructs

WSMV infectious cDNA clone (pSP6-WSMV) generated from isolate Sidney 81 [36] was used as a template for PCR amplification of P1 and all its mutants. Deletion mutants were named according to the number of residues deleted from the N- or C-terminal region. For example, N21 represents P1 cistron lacking 21 amino acid residues at the N-terminus. W303A point mutation was introduced in P1 cistron through site-directed mutagenesis by overlap extension PCR using primer W-525 (5’-GGATCACGAAGTGACGCTTGGAGCAAG TGGTGTTCTGCTTAGTG-3’) and its reverse compliment primer W-526. PCR-amplified P1 and its deletion or site-directed mutant sequences were inserted into pRTL2 [37] and then transferred into a binary vector pPZP212. pPZP212 with P1 sequences were chemically transformed into *Agrobacterium tumefaciens* strain EHA105. pWSMV-GFP-P1-W303A was created by introducing W303A point mutation in pSP6-WSMV-GFP-6K1/CI [38]. pPVX-WSMV-P1-W303A was a chimeric insertion of W303A mutation in the P1 cistron in PVX vector pP2C2S [39] between the *Cla*I and *Asc*I restriction sites. For protein expression in the bacterial system, P1 or P1-W303A cistrons were cloned between the *Nco*I and *Bam*HI restriction sites of pMAL-c5X and then chemically transformed into a NEB express strain of *Escherichia coli* (New England Biolabs Inc., Ipswich, MA, USA). Herculase II Fusion DNA polymerase (Agilent Technologies, Santa Clara, CA, USA) was used for all PCR reactions. The presence of introduced deletions or mutations in all constructs was verified by sequencing on Applied Biosystems 3730xl DNA Analyzer at the University of Florida ICBR Core DNA sequencing facility.

### 2.2. GFP Reporter Assays

Agrobacteria harboring P1 cistron or its mutants were grown overnight and resuspended in 10 mM MES, pH 5.45 containing 10 mM MgCl_2_ and 100 µM Acetosyringone to the optical density of 1.0 at 600 nm. This suspension was incubated at room temperature for ~3 h and mixed with 1.0 OD_600_ agrobacterial suspension of pPZP212-35S:GFP (35S:ssGFP) [40] and infiltrated into the abaxial side of fully expanded leaves of *N. benthamiana* at the 6–8 leaf stage. Plants were maintained in a growth chamber at 24–26 °C with a 14 h photoperiod. Agroinfiltrated leaves were observed for green fluorescence under long range UV light at 3 and 6 days post-agroinfiltration (dpa) and photographed with a Nikon D70 DSLR camera using an orange filter.

### 2.3. Northern Blot Hybridization

Total RNA was extracted from 400 mg of agroinfiltrated *N. benthamiana* leaf patches using the TriPure isolation reagent (Roche, Indianapolis, IN, USA). Two µg of total RNA per sample was electrophoresed in a 1% agarose-formaldehyde gel or 15% urea-polyacrylamide gel, followed by blotting onto a positively charged nylon membrane for detecting GFP mRNA or siRNAs, respectively, using GFP-specific digoxigenin (DIG)-labelled antisense riboprobes as described in Tatineni et al. [29].

### 2.4. Sequence Analysis

Multiple sequence alignment of select P1 sequences of potyvirid species was performed using deep substitution scoring matrix, BLOSUM62 [41]. GenBank sequences of P1 cistrons of different potyvirid species were translated and manually trimmed upon full-length multiple sequence alignment to highlight the domains of interest. Kyte–Doolittle hydropathy plot was generated using ExPASy ProtScale program [42]. Secondary structure prediction was performed using the Chou–Fasman method [43].

### 2.5. Inoculation of Plants

In vitro transcripts were synthesized from linearized pWSMV-GFP (pSP6-WSMV-GFP-6K1/CI) [38], pWSMV-GFP-P1-W303A, and pWSMV-GFP-ΔP1 [44] and mechanically inoculated onto fully expanded wheat seedlings at the single leaf stage as described in Tatineni et al. [38]. Inoculated wheat plants were incubated in a greenhouse at 22 to 27 °C with a 16 h photoperiod. Inoculated leaves at 10 days post-inoculation (dpi) and upper non-inoculated leaves at 21 dpi were collected for GFP fluorescence observation using a Zeiss Stereo Discovery V12 Fluorescence Microscope equipped with a narrow band GFP filter set 38 with 400–450 nm excitation and 450–490 nm emission. Fluorescent micrographs were captured using an AxioCam MRc5 camera attached to the fluorescent microscope. One µg of linearized plasmid DNA of pP2C2S-PVX [39], pPVX-WSMV-P1, or pPVX-WSMV-P1-W303A was transcribed in vitro and mechanically inoculated onto fully expanded *N. benthamiana* leaves at the six-leaf stage and incubated in a growth chamber at 25 °C with a 16 h photoperiod. 

### 2.6. RT-PCR

Total nucleic acids were isolated from wheat leaves inoculated with WSMV-GFP, WSMV-GFP-P1-W303A, or WSMV-GFP-ΔP1 by the glycine-phenol method as described in McNeil et al. [45]. One microgram of total nucleic acids was incubated with 25 ng of random hexamers and reverse transcribed into cDNA using the SuperScript III First-Strand Synthesis System (Invitrogen, Carlsbad, CA, USA), followed by subsequent PCR with primers XV1 and XC1 [46]. Total RNA was extracted from upper non-inoculated leaves of *N. benthamiana* plants inoculated with PVX, PVX-WSMV-P1, or PVX-WSMV-P1-W303A at 10 and 15 dpi using TriPure isolation reagent, followed by reverse transcription with random primers and PCR with primers Tr-206 and Tr-207 [44]. 

### 2.7. Real-Time RT-PCR

Real-time RT-PCR (RT-qPCR) was performed on total RNA extracted from PVX-infected upper symptomatic leaves collected at 10 and 15 dpi. Relative expression of PVX was determined on two biological replicates which were tested in duplicates by RT-qPCR with primers G-35 and G-36 [44] using SsoAdvanced SYBR Green Supermix (Bio-Rad) on Bio-Rad CFX Connect Real-Time PCR System. Thermocycler was set to initial denaturation for 2 min at 95 °C, followed by 40 cycles of 10 s denaturation at 95 °C, 30 s annealing at 55 °C, and 1 min extension at 72 °C. No-template and no-RT reactions were included as negative controls for PCR. The ΔΔCt method was used to compute relative expression of PVX genomic RNA with *N. benthamiana* actin as an internal reference gene for normalization. Non-specific expression from buffer-inoculated plants was subtracted from relative expression values from PVX-infected samples.

### 2.8. Western Blotting

Total proteins were extracted from inoculated and upper non-inoculated wheat leaves by homogenizing 0.4 g leaf pieces in 2 mL of TPE buffer (50 mM Tris-acetate pH 7.4, 10 mM potassium acetate, 1 mM EDTA, 5 mM DTT) containing 1 Complete Mini Protease Inhibitor Cocktail tablet (Roche, Indianapolis, IN, USA) per 10 mL of TPE buffer as described in Tatineni et al. [38]. The tissue lysate was mixed with an equal volume of 2× Laemmli buffer, followed by incubation in boiling water bath for 3 min and subjected to SDS-PAGE for immunoblotting with anti-GFP monoclonal antibody (Clontech, Mountain View, CA, USA) or WSMV CP specific polyclonal antibodies.

### 2.9. Electrophoretic Mobility Shift Assay

Maltose binding protein (MBP)-tagged proteins of WSMV P1 or P1-W303A were expressed in *E. coli* through pMAL-c5X and purified by affinity chromatography with amylose resin (New England Biolabs, Ipswich, MA, USA). Affinity purified proteins were quantified by the Bradford assay using Quick Start Bradford kit (Bio-Rad). PTGS-like ds-siRNAs were prepared from chemically synthesized 21- or 24-nt 5’P-ssRNA with 2-nt 3’ overhangs (Integrated DNA Technologies, Skokie IL) from GFP sequence. These ds-siRNAs were diluted to 1 µM concentration in 20 mM Tris-HCl (pH 8.0), 1 mM EDTA and 50 mM NaCl (TEN buffer). Long dsRNA was prepared by annealing in vitro transcribed positive- and negative-sense ssRNAs derived from GFP (180-nt) or WSMV CP (600-nt) sequences in TEN-T buffer (TEN buffer containing 0.02% Tween 20). 

Synthetic ds-siRNAs or ss/dsRNA (100 ng) was incubated with MBP-tagged protein (0.3 nmol) in 30 µL of TEN buffer for 30 min at room temperature. Following incubation, the reaction mixture was analyzed through 4–12% non-denaturing polyacrylamide gel electrophoresis at 4 °C and visualized with SYBR Green stain (Thermo Scientific, Waltham, MA, USA) as described in Samuel et al. [47]. ImageJ software was used to quantify the fluorescent signal of unbound dsRNA bands [48].

### 2.10. Dicer Protection Assay

In vitro dicing reaction was performed by incubating 0.3 nmol of affinity purified MBP-tagged WSMV P1 or MBP and 1.0 µg of 600-nt dsRNA with 0.5 U of recombinant human Dicer enzyme (Turbo Dicer siRNA generation kit, Genlantis, San Diego, CA, USA) in a 30 µL reaction mixture of 250 mM NaCl, 30 mM HEPES (pH 8.0), 0.05 mM EDTA, 2.5 mM MgCl_2_ and 1 mM ATP. The Dicer reaction mixture was incubated overnight at 37 °C. Following incubation, the reaction mix was quenched with 10 mM EDTA, resolved on a 4–12% non-denaturing polyacrylamide gel at 4 °C, and visualized by staining with SYBR Green. In the dicing reaction, MBP was used as a negative control for MBP tags and for non-dsRNA binding protein.

## 3. Results

### 3.1. The N-terminal 25 Amino Acids but not a Single C-terminal Amino Acid of WSMV P1 Are Dispensable for VSR Activity

**Figure 1:**
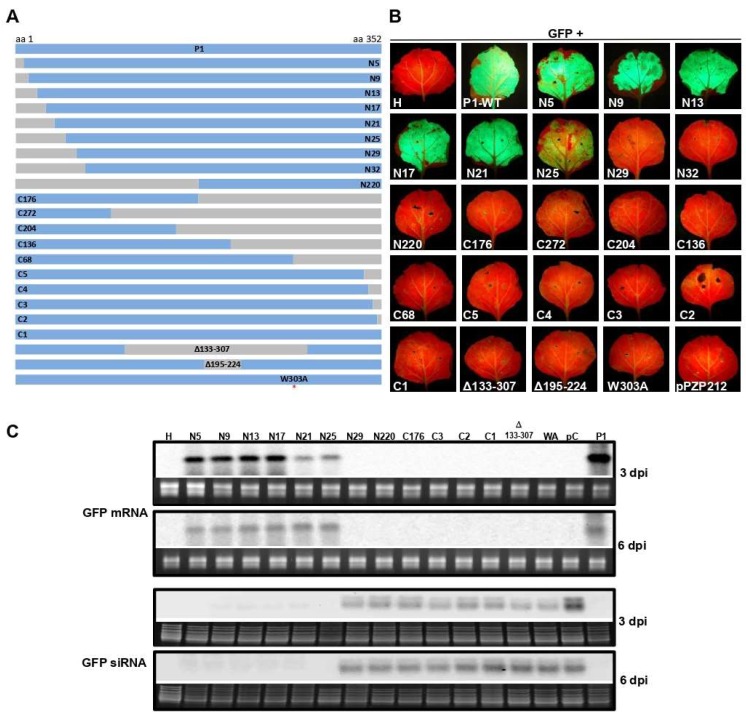
Analyses of *wheat streak mosaic virus* (WSMV) P1 for RNA silencing suppression activity. (**A**) Map of deletion and site-directed mutants of WSMV P1 engineered under a CaMV 35S promoter in pPZP212 for agroinfiltration into *Nicotiana benthamiana* leaves for GFP reporter assay. (**B**) Green fluorescent images of *N. benthamiana* leaves agroinfiltrated with indicated constructs along with pPZP212-35S:GFP as a reporter at 6 days postagroinfiltration (dpa). “H” indicates uninfiltrated healthy leaf; pPZP212 indicates agroinfiltration of pPZP212 along with pPZP212-35S:GFP (negative control). Note the loss of VSR activity of WSMV P1 with disrupted GW motif (W303A). (**C**) Northern blot hybridization of total RNA extracted from agroinfiltrated regions of selected constructs as indicated above the lanes. The top two and the bottom two panels show accumulation of GFP mRNA and siRNAs at 3 and 6 dpa, respectively. Ethidium bromide stained gel images shown below the Northern blots of GFP mRNA and GFP siRNAs are rRNA and low molecular weight RNAs, respectively, for the amount of RNA loaded per lane.

To examine the WSMV P1 sequence (352 amino acids) harboring functional domains for VSR function, progressive deletions were introduced from the N- and C-terminal regions of P1, followed by agroinfiltration-based GFP reporter assay. *Agrobacterium*-harboring pPZP212 with P1 that contained nine and ten progressive deletions from the N- and C-terminal regions, respectively, were co-infiltrated with 35S:ssGFP reporter into the leaves of *N. benthamiana* at the 6–8 leaf stage (Figure 1A).

Prolonged green fluorescence was observed at 6 dpa in *N. benthamiana* leaves infiltrated with N-terminal deletion of 25 amino acids or less. In contrast, none of the C-terminal deletion mutants facilitated prolonged expression of GFP (Figure 1B). Northern blot was performed on total RNA extracted from infiltrated regions of *N. benthamiana* leaves at 3 and 6 dpa to examine the extent of silencing and silencing suppression of GFP reporter by examining the accumulation of GFP specific siRNAs and mRNA, respectively. Northern blot hybridization revealed that GFP-specific mRNA accumulated in agroinfiltrated leaf patches with P1 containing deletion of 25 N-terminal or less amino acids but not more than 29 amino acids at 3 and 6 dpi (Figure 1C). By contrast, GFP mRNA did not accumulate at detectable levels in agroinfiltrated leaves with P1 harboring deletions at the C-terminal and internal regions (Figure 1C). In agroinfiltrated leaf patches, GFP-specific siRNAs accumulated at enhanced levels with P1 deletion mutants that failed to accumulate GFP mRNA, similar to that of pPZP212 control (Figure 1C). These results revealed that the 25 N-terminal amino acids of WSMV P1 are dispensable for suppression of RNA silencing induced by ssRNA.

### 3.2. WSMV P1 Binds to dsRNA and Sequesters PTGS-like ds-siRNAs

**Figure 2:**
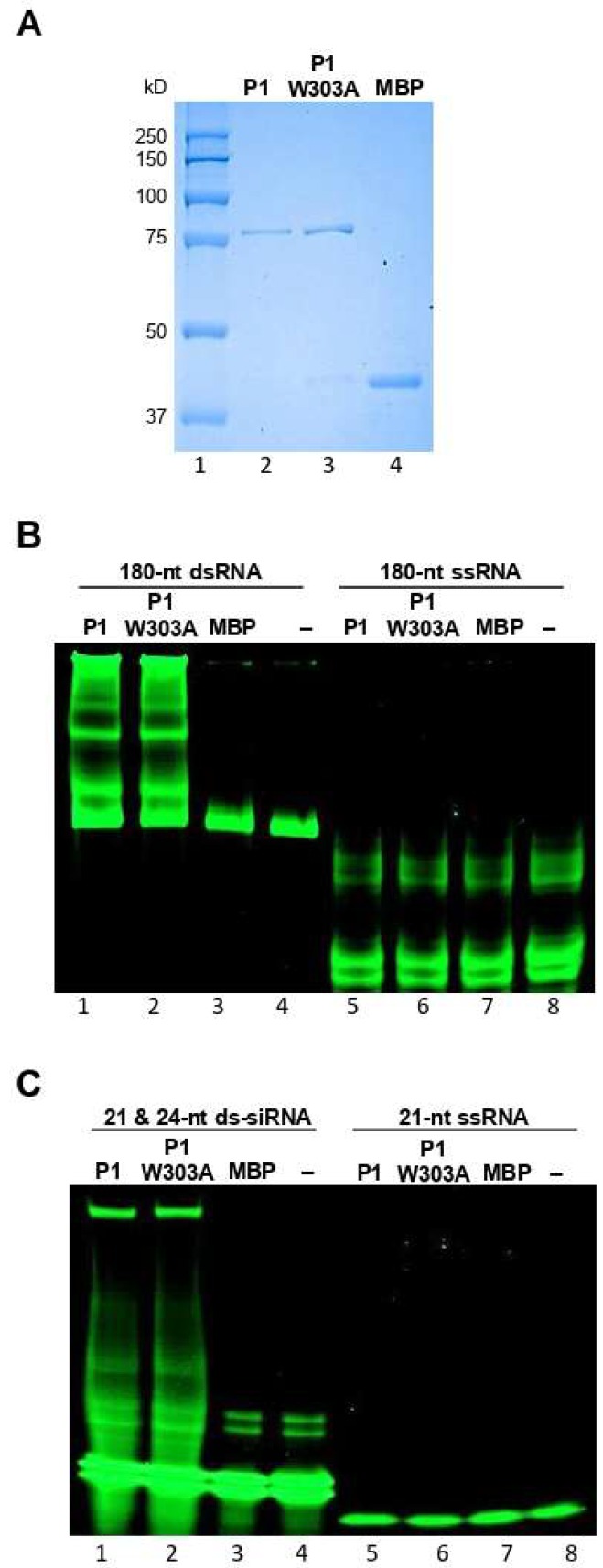
WSMV P1 interacts with dsRNA with no size specificity. (**A**) SDS-PAGE showing the affinity purified MBP::P1 (lane 2), MBP::P1-W303A (lane 3) and MBP (lane 4) expressed in *E. coli* and stained with Coomassie Brilliant Blue R-250. Lane 1 represents protein standard. (**B**) Electrophoretic mobility shift assay of WSMV P1 binding to 180-nt ds- (lane 1–4) and ssRNAs (lanes 5–8) derived from GFP. 180-nt dsRNA formed complexes with WSMV P1 (lane 1) and P1-W303A (lane 2) as a shift in electrophoretic mobility, but not with 180-nt ssRNA (lanes 5 and 6). (**C**) Electrophoretic mobility shift assay of WSMV P1 binding to 21- and 24-nt ds-siRNAs (lanes 1–4) and 21-nt ssRNA (lanes 5–8). 21- and 24-nt ds-siRNAs formed complexes with WSMV P1 and P1-W303A (lanes 1 and 2) but not with 21-nt ssRNA (lanes 5 and 6). RNAs incubated with proteins were separated through 4–12% non-denaturing polyacrylamide gel electrophoresis, followed by SYBR Green staining as described in Samuel et al. [47]. MBP included as an unrelated protein control as well as a control for MBP affinity tags of pMAL expressed P1 and P1-W303A proteins.

We next examined the mechanisms of suppression of RNA silencing by WSMV P1. As the first step, the dsRNA binding property of P1 was examined by performing electrophoretic mobility shift assays. The P1 cistron was engineered into pMAL-c5X, followed by in vitro expression in *E. coli* as MBP fusion protein, MBP::P1 (Figure 2A, lane 2). The MBP::P1 protein was incubated with in vitro synthesized GFP-specific 180-nt dsRNA or 21- and 24-nt ds-siRNAs. The extent of protein-RNA interactions was examined through non-denaturing PAGE, followed by SYBR Green staining. The MBP::P1, but not MBP, formed complexes with 180-nt dsRNA as revealed by electrophoretic mobility shift in comparison with RNA-only control (Figure 2B, compare lane 1 with lanes 3 and 4). MBP::P1 also exhibited a strong interaction with 21- and 24-nt ds-siRNAs as evidenced by a shift in electrophoretic mobility compared with MBP or ds-siRNA-only controls (Figure 2C, compare lane 1 with lanes 3 and 4). These data suggest that WSMV P1 binds to dsRNAs of 180-nt, 21-nt, and 24-nt of GFP origin. We next examined whether WSMV P1 binds to ssRNA by incubating MBP::P1 with GFP-specific 180-nt or 21-nt ssRNA, followed by PAGE separation. Our data revealed that MBP::P1 did not form complexes with 180-nt or 21-nt ssRNAs from GFP at detectable levels (Figure 2B,C, compare lanes 5 with lanes 7 and 8).

**Figure 3:**
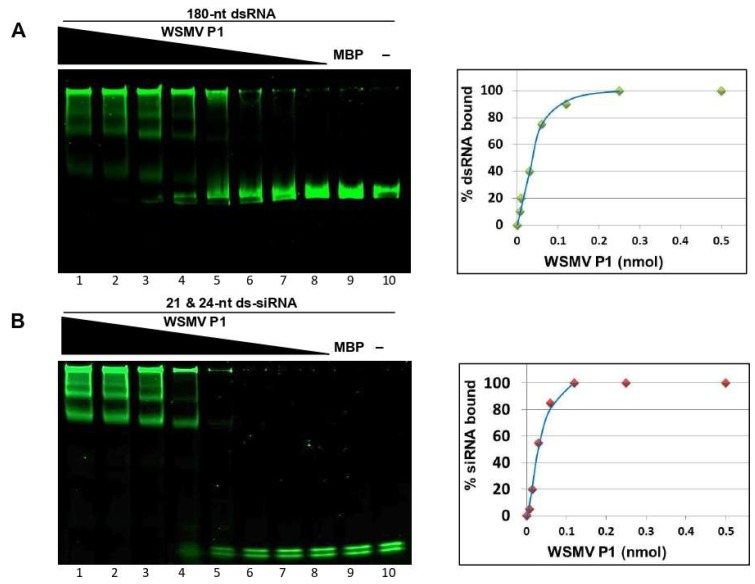
Dynamics of WSMV P1 and dsRNA interaction. WSMV P1 in a two-fold serial dilution starting from 0.5 nmol (lanes 1–8) or MBP (lane 9) incubated with 100 ng of 180-nt dsRNA (**A**) or 50 ng of 21- and 24-nt ds-siRNAs (**B**). RNA-only lane is represented as “−“ (lane 10). Lower bands represent unbound dsRNA, whose intensities of fluorescence subtracted from 100% (total dsRNA loaded) were plotted against the amount of MBP::P1 loaded. RNAs incubated with serially diluted MBP::P1 or with 0.5 nmol of MBP were separated through 4–12% non-denaturing polyacrylamide gel electrophoresis, followed by SYBR Green staining. The amount of fluorescence in lower bands represent percentage of unbound RNA whose differences from the amount of RNA used initially (100%) were plotted against the amount of MBP::P1 loaded in each well (right).

The stoichiometry of VSR-dsRNA interaction inferred from binding kinetics is significant for determining dsRNA affinity of VSR, signifying the functional importance of VSR efficacy [49]. Additionally, binding kinetics also reveal dsRNA size preference of VSR. The binding kinetics of P1 were performed by incubating two-fold serially diluted MBP::P1 with a standard amount of 180-nt dsRNA or 21- and 24-nt ds-siRNAs, followed by non-denaturing PAGE. The percentage of dsRNA binding at a given P1 concentration was calculated by subtracting the unbound fraction of dsRNA from the amount of dsRNA used initially (100%) for binding reaction. These values were plotted against corresponding amount of MBP::P1 used in the binding reaction to result in an affinity curve for 180-nt dsRNA (Figure 3A) or 21- and 24-nt ds-siRNAs (Figure 3B). The magnitude of binding affinity for each dsRNA species is represented in the skewness of its respective curve [50,51]. Electrophoretic mobility shift assay analyses and affinity curves suggested that WSMV P1 binds to 180-nt dsRNA and 21- and 24-nt ds-siRNAs in a dose dependent manner with no preference for size. Affinity curves derived from binding kinetics revealed that ~0.03 nmol of MBP::P1 was required to bind ~50 % of 180-nt dsRNA or 21- and 24-nt ds-siRNAs used for binding assays. These data suggest that WSMV P1 possess strong dsRNA binding property.

### 3.3. P1 Inhibits Dicing of Long dsRNA

**Figure 4:**
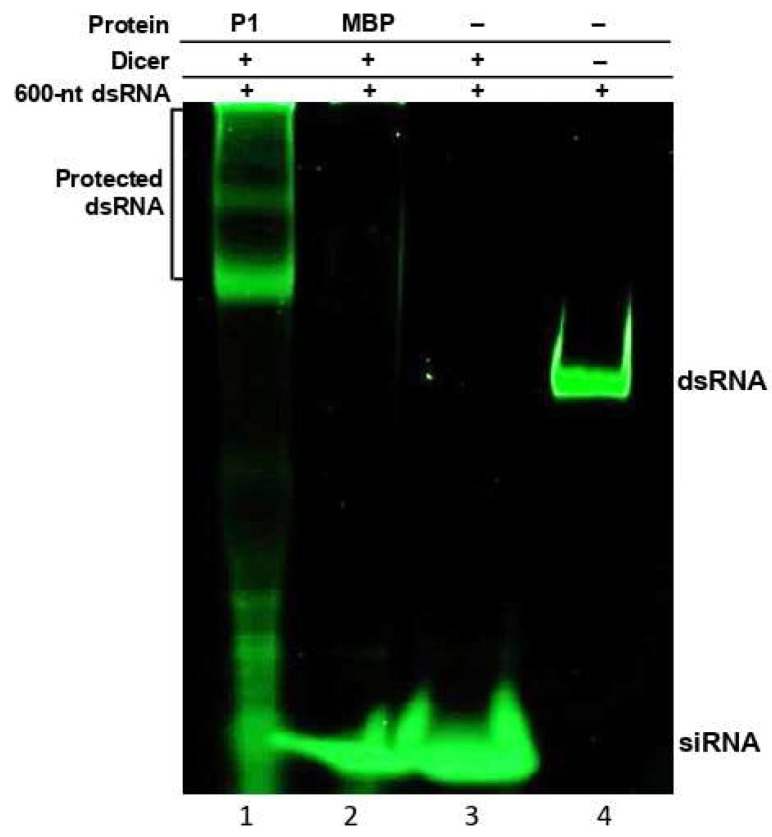
WSMV P1 protects dsRNA from dicing in vitro. In vitro dicing reaction was performed by incubating 600-nt dsRNA (100 ng) from WSMV coat protein sequence with recombinant human Dicer plus MBP-tagged WSMV P1 (lane 1) or MBP (lane 2). Lane 3: no protein control; lane 4: RNA-only control (lane 4). MBP was used as a non-viral suppressor of RNA silencing protein as well as a control for MBP affinity tag of MBP::P1. Besides protection against Dicer, shift in electrophoretic mobility of dsRNA in the presence of P1 was observed. RNAs incubated in dicing reactions were separated through 4–12% non-denaturing polyacrylamide gel electrophoresis, followed by SYBR Green staining.

Electrophoretic mobility shift assays revealed that WSMV P1 binds to dsRNAs with no size preference. This property might be significant in protecting viral dsRNA from the activity of DCLs in RNA silencing, thereby hampering or delaying siRNA generation. To determine whether dsRNA binding property of P1 has any impact on DCL activity, in vitro dicing reactions were performed using recombinant human Dicer on 600-nt dsRNA derived from WSMV CP cistron in the presence of MBP::P1 (Figure 4).

When MBP::P1 was supplemented with Dicer enzyme in a dicing reaction, the P1 protein protected long dsRNA from being diced into siRNAs by forming P1-dsRNA complexes (Figure 4, lane 1). The binding of long dsRNA to MBP::P1 resulted in an electrophoretic mobility shift with a remarkable protection of dsRNA from Dicer mediated hydrolysis. However, the degraded fraction of unbound dsRNA was visualized as shorter dsRNA molecules and traces of siRNA accumulation. In dicing reactions supplemented with MBP or no protein, siRNA accumulation was observed with little or no detectable intact dsRNA (Figure 4, lanes 2 and 3), suggesting Dicer-mediated hydrolysis. These results are consistent with the affinity of P1 toward long dsRNA, thus placing a strong hurdle in the RNA silencing pathway by blocking generation of siRNAs. Additionally, these data revealed that WSMV P1 equally binds to GFP- and CP-specific dsRNAs in electrophoretic mobility shift and the Dicer assays, respectively, suggesting that P1 binds to dsRNA with no sequence specificity.

### 3.4. GW Motif of WSMV P1 Is Essential for Suppression of ssRNA Induced Local Silencing

**Figure 5:**
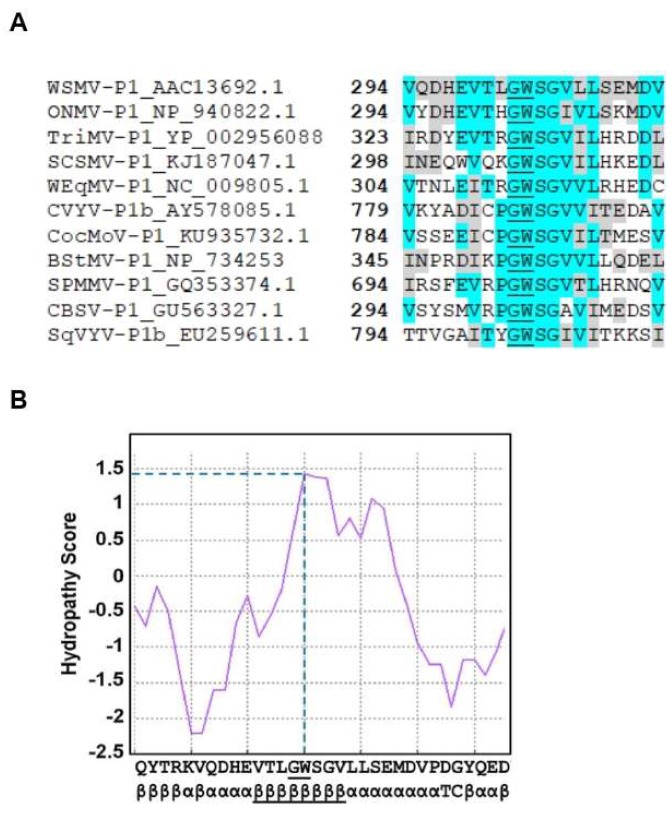
WSMV P1 harbors a conserved GW motif. (**A**) Multiple sequence alignment of GW containing AGO interacting domains of P1-like proteins of *Wheat streak mosaic tritimovirus* (WSMV), *Oat necrotic mottle tritimovirus* (ONMV), *Triticum mosaic poacevirus* (TriMV), *Sugarcane streak mosaic poacevirus* (SCSMV), *Wheat eqlid mosaic tritimovirus* (WEqMV), *Cucumber vein yellowing ipomovirus* (CVYV), *Coccinia mottle ipomovirus* (CocMoV), *Brome streak mosaic tritimovirus* (BStMV), *Sweet potato mild mottle ipomovirus* (SPMMV), *Cassava brown streak ipomovirus* (CBSV), and *Squash vein yellowing ipomovirus* (SqVYV). GenBank accession number for each sequence was indicated in the label of corresponding sequence. The numbers in bold indicate the position of first residue of each sequence within its cistron. A GW dipeptide motif was underlined within each sequence. Blocks highlighted in turquoise indicate similar amino acids with high sequence conservation, while blocks highlighted in gray indicate amino acids with similar biochemical properties. (**B**) Hydropathy analysis and secondary structure prediction of partial sequence of WSMV P1 (aa 289–320) indicates high accessibility of GW dipeptide in the cellular environment. GW dipeptide and its whole β-sheet were underlined to indicate their relative positions in the sequence.

Deletion analysis indicated that P1 did not tolerate deletion of a single amino acid at the C-terminus for RNA silencing suppression activity. These results suggested the presence of an important domain at the C-terminal region of P1 that might be required for VSR function. Sequence analysis of WSMV P1 revealed the presence of a single glycine-tryptophan (GW) dipeptide motif toward the C-terminal region, along with few adjacent amino acids, that showed high sequence homology with few other P1-like proteins of potyvirid species (Figure 5A). Additionally, computational analyses of GW-motif containing domain by Kyte–Doolittle method showed hydropathy score of 1.40 (Figure 5B), which suggested extensive solubility. An ipomoviral P1 was shown to inhibit AGO1 through three N-terminal WG/GW motifs [19]. However, WSMV P1 lacks the N-terminal WG/GW motifs but has a single C-terminal GW motif.

To determine whether the GW motif is required for silencing suppression activity of WSMV P1, a site-directed mutant, pCASS4-WSMV-P1-W303A (35S:P1-W303A), was generated (Figure 1A; red asterisk). Agroinfiltration of 35S:P1-W303A and 35S:ssGFP reporter construct into *N. benthamiana* leaves at the 6–8 leaf stage revealed no expression of GFP at 3 and 6 dpa (Figure 1B). Northern blot analysis of total RNA extracted from infiltrated leaf patches revealed no detectable levels of GFP mRNA while GFP-specific siRNAs accumulated at readily detectable levels (Figure 1C). These data suggest that GW motif of WSMV P1 is essential for suppression of local silencing induced by ssRNA.

### 3.5. Mutagenesis of GW Motif Does not Disrupt dsRNA Binding Properties

The above experiments indicated that the GW motif in WSMV P1 is required for VSR function. To determine whether the loss of silencing suppression function of P1-W303A mutant was due to loss of dsRNA binding activity, we performed in vitro dsRNA binding assays with MBP::P1-W303A. MBP-tagged P1-W303A protein was expressed in *E. coli* (Figure 2A, lane 3) and incubated with 180-nt dsRNA or 21- and 24-nt ds-siRNAs. Following non-denature PAGE analysis, dsRNA or ds-siRNAs incubated with MBP::P1-W303A formed complexes with retarded electrophoretic mobility similar to that of MBP::P1 (Figure 2B,C, compare lanes 2 with lanes 1), suggesting that disruption of GW motif did not affect dsRNA binding properties of WSMV P1. Taken together, these data revealed that dsRNA binding is not the only mechanism of WSMV P1 that enables suppression of RNA silencing.

### 3.6. WSMV P1-W303A Fails to Enhance Pathogenicity of a Heterologous Virus

**Figure 6:**
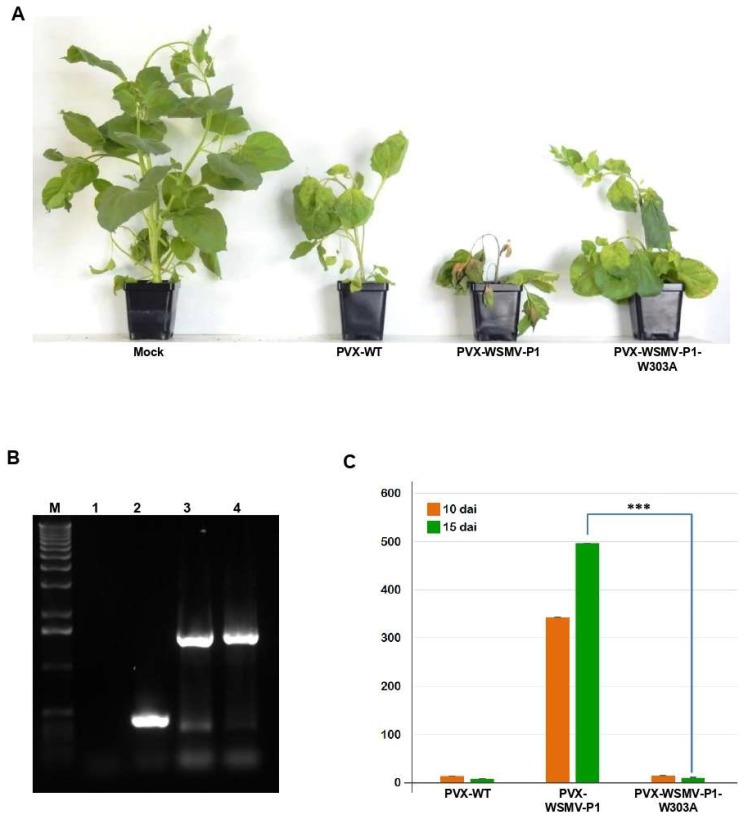
GW motif in WSMV P1 is required for pathogenicity enhancement of *Potato virus X* (PVX) in *Nicotiana benthamiana*. (**A**) Symptoms elicited by wild-type and chimeric PVX on *N. benthamiana* at 15 dpi. Note PVX-WSMV-P1 elicited severe symptoms while PVX-WSMV-P1-W303A elicited mild symptoms similar to PVX-WT. (**B**) RT-PCR analysis of total RNA isolated from symptomatic leaves (15 dpi) of *N. benthamiana* plants inoculated with PVX-WT (lane 2), PVX-WSMV-P1 (lane 3) or PVX-WSMV-P1-W303A (lane 4) with primers flanking the site of insertion. Lane 1: mock-inoculated *N. benthamiana*. Lane M represents 1.0 kbp DNA size standard. (**C**) Real-time RT-PCR analysis of total RNA extracted from symptomatic *N. benthamiana* leaves to determine the relative accumulation of PVX. The ΔΔCt method was used to determine the relative expression of PVX upon normalization with Nb-Actin as an internal reference. Student’s *T*-Test was conducted to calculate the most probable differences of PVX relative expression in each biological replicate with 99% confidence represented as ***. Bars on histograms indicate standard error.

Viral suppressors of RNA silencing are known to be inducers of pathogenicity in host plants [29,44,52,53,54]. Previously, WSMV P1 has been shown to enhance disease severity of PVX in *N. benthamiana* [26]. Here, the requirement of GW-motif for enhancement of pathogenicity was examined by inserting WSMV P1 cistron with W303A mutation into PVX [39]. *N. benthamiana* plants inoculated with in vitro transcripts of PVX-P1-W303A showed inward curling of upper leaves with mild mosaic symptoms similar to those elicited by PVX-WSMV-P1 and PVX-WT at 8 dpi. At 15 dpi, plants inoculated with PVX-WSMV-P1 elicited severe stunting and apical necrosis (Figure 6A). In contrast, PVX-WSMV-P1-W303A elicited mild mosaic symptoms due to recovery and resumption of apical growth similar to that of wild-type PVX, suggesting that the GW motif is critical for enhanced pathogenicity induced by P1 (Figure 6A). Stability of P1 or P1-W303A sequences in PVX in *N. benthamiana* plants was confirmed by RT-PCR on total RNA extracted from symptomatic leaves at 15 dpi with primers flanking the site of insertion, and found that the inserted sequences were stably maintained in PVX (Figure 6B). 

We next examined the accumulation of PVX from PVX-, PVX-WSMV-P1-, or PVX-WSMV-P1-W303A-infected *N. benthamiana* plants at 10 and 15 dpi by RT-qPCR. Upon normalization with expression of Nb-Actin as a reference gene, accumulation of PVX-WSMV-P1-W303A was 25- and 60-fold less at 10 and 15 dpi, respectively, compared with PVX-WSMV-P1. Moreover, relative expression of PVX-WSMV-P1-W303A was similar to that of PVX-WT (Figure 6C). These results indicated that disruption of GW motif in P1 not only resulted in loss of RNA silencing suppression function, but also its ability to enhance virulence.

### 3.7. Disruption of GW Motif in P1 Cistron Is Lethal to WSMV

**Figure 7:**
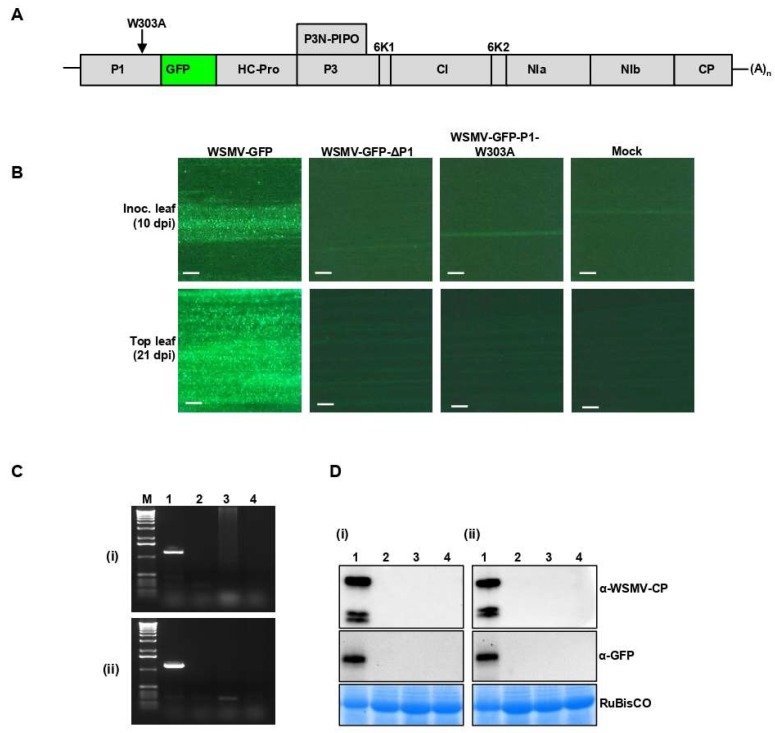
Disruption of GW motif in WSMV P1 led to the loss of WSMV viability. (**A**) Schematic representation of WSMV-GFP genome showing the position of W303A mutation. (**B**) Fluorescent micrographs showing the local foci (top panel) and systemic infection (bottom panel) on wheat inoculated with in vitro transcripts of WSMV-GFP, WSMV-GFP-ΔP1, or WSMV-GFP-P1-W303A. Mock: inoculated with buffer. The scale bars represent 200 µm. (**C**) RT-PCR of WSMV CP performed on total RNA prepared from inoculated leaves at 10 dpi (i) or in upper non-inoculated leaves at 21 dpi (ii) from WSMV-GFP (lane 1), WSMV-GFP-ΔP1 (lane 2), WSMV-GFP-P1-W303A (lane 3), and buffer (mock, lane 4) inoculated wheat plants. Lane M represents 1.0 kbp DNA marker. (**D**) Western blot analysis of total proteins extracted from local inoculated leaves (i) or from upper non-inoculated leaves (ii) to detect WSMV CP (top panel) or GFP (middle panel) from WSMV-GFP (lane 1), WSMV-GFP-ΔP1 (lane 2), WSMV-GFP-P1-W303A (lane 3), or buffer (mock, lane 4) inoculated wheat plants. Coomassie Brilliant Blue R-250 stained SDS-PAGE show wheat RuBisCO protein for the amount of protein loaded per well (bottom panel).

To test whether disruption of GW motif in P1 cistron affects the viability of WSMV in terms of cell-to-cell or long-distance movement, W303A site-directed mutation was introduced in P1 cistron in WSMV-GFP to generate WSMV-GFP-P1-W303A (Figure 7A). Capped in vitro transcripts of WSMV-GFP-P1-W303A were mechanically inoculated onto 15–20 wheat seedlings at the single-leaf stage. WSMV-GFP and P1-deficient WSMV-GFP (WSMV-GFP-ΔP1) [44] were used as positive and negative controls, respectively. 

Examining wheat leaves under the fluorescent microscope revealed formation of local foci at 10 dpi only in wheat seedlings inoculated with WSMV-GFP but not from WSMV lacking the P1 or P1 with W303A mutation (Figure 7B). At 21 dpi, 90–100% of wheat seedlings inoculated with WSMV-GFP showed mosaic, mottling, and chlorotic streak symptoms. However, wheat seedlings inoculated with WSMV-GFP-P1-W303A elicited no symptoms similar to that of WSMV-GFP-ΔP1 or buffer inoculated seedlings. RT-PCR was performed on total RNA isolated from inoculated (at 10 dpi) and upper non-inoculated leaves (at 21 dpi) using primers targeting WSMV CP. PCR amplification was obtained only from WSMV-GFP-inoculated wheat (Figure 7C). Similarly, Western blot analyses of total proteins from inoculated and upper non-inoculated leaves detected CP and GFP in wheat inoculated with WSMV-GFP but not with WSMV-GFP-P1-W303A- or WSMV-GFP-ΔP1 (Figure 7D). Collectively, these data suggest that the GW motif of P1 is required for the viability of WSMV.

## 4. Discussion

Due to their obligate parasitic nature, viruses face several challenges in not only utilizing the host cellular resources but also overwhelming host biology to establish systemic infection. In the evolutionary tug-of-war between host and virus, viruses acquired suppressors of RNA silencing to overcome the host-induced defensive RNA silencing [11,24]. In this study, the mechanisms of WSMV P1 in suppression of RNA silencing were examined. We found that P1 binds to dsRNAs, but not to ssRNAs, without size and sequence specificity, and harbors a GW motif at the C-terminal region that is required for suppression of RNA silencing and enhancement of pathogenicity. Deletion analysis of P1 facilitated the identification of functional domains that are required for VSR function. Deletion of 25 N-terminal amino acids of P1 resulted in weak suppression of RNA silencing, but deletion of a single amino acid at the C-terminus completely abolished suppression of RNA silencing.

In order to examine the mechanisms of WSMV P1 suppression of RNA silencing, the P1 protein was expressed in vitro as MBP-fusion protein to enhance solubility [55]. Computational analyses of P1-like sequences of potyvirid species revealed the presence of a conserved siRNA binding domain [56] between amino acids 49 and 90 of WSMV P1 (Figure S1). Additionally, the conformational changes may be required in adjacent amino acids for siRNA sequestration [57,58], which could potentially occur between amino acids 25 and 49. Though siRNA binding domain of CYVY P1b [56] showed reasonable conservation with WSMV P1, several charged and polar amino acids showed substitutions with amino acids of similar biochemical properties, suggesting that conservation of structural features [59] required for siRNA binding. Valli et al. [56] showed that mutation of few basic amino acid residues in P1b of CVYV resulted in loss of ds-siRNA binding activity, suggesting an important role for these amino acids in VSR function.

Binding of WSMV P1 to dsRNAs in a size-independent manner is considered as its role in silencing suppression at two different stages: i. Protection of dsRNAs from dicing, which would dramatically reduce siRNA generation, and ii. Sequestration of ds-siRNAs from the host defense system would potentially create an impediment for RISC formation [12]. WSMV P1 interaction with dsRNA or ds-siRNAs formed complexes that resulted in multiple bands, suggesting that several molecules of protein are involved in association and dissociation with each RNA molecule at any given time during incubation [60]. However, multiple bands observed in ssRNA incubated with P1 are due to the formation of stochastic secondary structures (unlike dsRNA), assuming differential electrophoretic properties for different molecules of the same transcript under non-denaturing conditions. The dsRNA binding property of P1 was tested for its functional significance by supplementing P1 in in vitro dicing of long dsRNA. P1 exhibited extensive protection of dsRNA from dicing, suggesting inaccessibility of dsRNA to human Dicer through P1-dsRNA interaction. The ability of WSMV P1 to bind 180-nt dsRNA from GFP as well as 600-nt dsRNA from WSMV CP suggest that WSMV P1 binds to dsRNA molecules in a sequence-independent manner.

Sequence analysis of the C-terminal region of WSMV P1 and other potyvirid P1-like sequences revealed high conservation, suggesting functional conservation among P1-like sequences with suppression of RNA silencing function. Loss of VSR function upon deletion of a single amino acid at the C-terminus emphasized the presence of a highly conserved C-terminal domain in WSMV P1 that could function in suppression of RNA silencing. Sequence analyses showed the presence of a conserved GW dipeptide at the C-terminal region. The GW182 family of proteins is known to interact with AGO-PIWI of miRISC to aid in target hydrolysis [61]. The GW dipeptide in other viral-encoded proteins demonstrated a role in suppression of RNA silencing through interaction with AGOs [19,62,63]. Besides AGO interaction, the GW motif of a silencing suppressor could also compete with GW182 proteins required for endonuclease activity of PIWI. Hydropathy analysis by using Kyte–Doolittle method showed hydrophilicity of the tryptophan residue [42]. Both the hydrophilicity and relative position of tryptophan residue suggested high solubility of GW motif and spatial conformation required for efficient interaction with AGOs [64,65].

Mutagenesis of W303A to disrupt the GW motif of WSMV P1 abolished silencing suppression function, pathogenicity enhancement of a heterologous virus, and replication and movement of WSMV. Loss of GFP expression in the presence of P1-W303A plus a GFP reporter construct in agroinfiltrated *N. benthamiana* leaves was due to loss of ability to suppress RNA silencing. Chimeric expression of a VSR has been shown to increase pathogenicity of a heterologous virus through its pathogenicity enhancement property [29,44,65], which was tested with WSMV P1 in PVX background on *N. benthamiana* [26]. However, infection of *N. benthamiana* plants by PVX-WSMV-P1-W303A elicited symptoms similar to wild-type PVX. Further evidence demonstrating the significance of GW motif of P1 in the virus life-cycle was the inability of mutant WSMV-GFP-P1-W303A to form viral foci or establish systemic infection in wheat. Suppression of host defensive RNA silencing is imperative for systemic infection of plants, which is possible only in the presence of a bona fide VSR. However, we cannot exclude mutation of W303A might have affected the cleavage of P1. The mutant P1-W303A protein did not lose its ability to bind to dsRNAs but lost the VSR function in a GFP reporter assay. This could be because interaction of P1 with other host-encoded RNA silencing proteins is indispensable for suppression of RNA silencing irrespective of P1’s ability to bind dsRNA for protection from Dicer and siRNA sequestration.

Viral suppressors of RNA silencing play a central role in combating the host defense systems. Studying suppressors of RNA silencing and unveiling their molecular mechanisms are important not only to understand their diversity, regulation, and evolution, but also to develop and fine-tune approaches for disease management. Conclusively, WSMV P1 suppresses host RNA silencing at multiple levels by protecting dsRNA from dicing, sequestering ds-siRNAs, and potentially interacting with Argonaute through GW motif to interfere with RISC formation and target hydrolysis. 

## Supplementary Materials

The following are available online at https://www.mdpi.com/1999-4915/11/5/472/s1, Figure S1: Multiple sequence alignment of putative dsRNA binding domains of P1-like proteins of *Wheat streak mosaic tritimovirus* (WSMV), *Oat necrotic mottle tritimovirus* (ONMV), *Cucumber vein yellowing ipomovirus* (CVYV), *Sugarcane streak mosaic poacevirus* (SCSMV), *Sweet potato mild mottle ipomovirus* (SPMMV), *Squash vein yellowing ipomovirus* (SqVYV), *Triticum mosaic poacevirus* (TriMV), *Wheat eqlid mosaic tritimovirus* (WEqMV), *Brome streak mosaic tritimovirus* (BStMV), and *Cassava brown streak ipomovirus* (CBSV). GenBank accession number for each sequence was indicated in the label of corresponding sequence along with the name of the viral cistron separated by an underscore. Bold faced numbers indicate the position of first residue of each sequence within its cistron. Blocks highlighted in turquoise indicate similar amino acids with high conservation, while blocks highlighted in gray indicate amino acids with similar biochemical properties. All sequences were conceptually translated and manually trimmed to highlight domains of interest.
