# Supplementary material for: Wheat streak mosaic virus P1 Binds to dsRNAs without Size and Sequence Specificity and a GW Motif Is Crucial for Suppression of RNA Silencing
Source: Viruses. 2019 May 24;11(5):472. doi: 10.3390/v11050472 (PMC6563293; doi:10.3390/v11050472)
Supplement: Supplementary file 1 [file viruses-11-00472-s001.pdf]

```
WSMV-P1_AAC13692.1        49  GSIFWSKEGILTQTAKNLYKATAYGLGYDLAANVFVCGKCRS
ONMV-P1_NP_940822.1       49  GSIFWSKDGVRTQTSKNLYKAMSLGLGYDLAADVFVCGMCRS
CVYV-P1b_AY578085.1      574  GGLVRNASGVTKPALACLRKATKYGVGFDMMMNHYVCCRCHV
SCSMV-P1_KJ187047.1       50  GSVFWTTKGKPKTIVNNLFKATQYGLAYDIAAEVYVCPICMT
SPMMV-P1_GQ353374.1      481  GGIYRTRKGSYKNAALRLLKATKVQVFYDGIKDIFECPYCHV
SqVYV-P1b_EU259611.1     590  GGIFRNQSGVYKNPAIMLRRAARYGLAFDCALEAYECPMCGM
TriMV-P1_YP_002956088     74  GSIFWDTNGRIKPVVNCLLRATRMNLDYDVAADVYVCRDCLS
WEqMV-P1_NC_009805.1      56  GSIFWSTDGKLTQTAKNVYLATDKKLGYDLAAELFVCGSCRS
BStMV-P1_NP_734253        99  GSIFKTRTGKDTPIATAIRAATRRGLAYDIAAQLYMCPKCCS
CBSV-P1_GU563327.1        88  GGVFKNKKGQELQAAKRLRRATSYGFMYDPVLKAFECPKCRT
```

Fig. S1
